# Aspirin Desensitization and Percutaneous Coronary Intervention in a Patient with Aspirin Hypersensitivity and Acute Coronary Syndrome: A Case Report

**DOI:** 10.31729/jnma.8643

**Published:** 2024-07-31

**Authors:** Siddinath Gyawali, Suman Acharya, Sanjeev Kharel, Dinesh Upreti, Khem Raj Bhusal, Silvia Maharjan, Hemanta Shrestha, Ratna Mani Gajurel

**Affiliations:** 1 Department of Internal Medicine, Tribhuvan University Teaching Hospital, Institute of Medicine, Kathmandu, Nepal.; 2 Department of Cardiology, Manmohan Cardiothoracic Vascular and Transplant Center, Institute of Medicine, Kathmandu, Nepal

**Keywords:** *acute coronary syndrome*, *aspirin*, *case report*, *coronary angioplasty*, *hypersensitivity*

## Abstract

Hypersensitivity to aspirin is rare disorder occurring in 1.88% of the patients. Aspirin-hypersensitive patients requiring single antiplatelet agent may be treated with clopidogrel, an alternative antiplatelet agent. However, aspirin desensitization is more cost-effective than the usage of clopidogrel in these patients. Furthermore, aspirin desensitization is of greater value in patients requiring dual antiplatelet therapy, for example following procedures like percutaneous transluminal coronary angioplasty (PTCA) instead of using non-aspirin-based combinations. Herein, we report a 74-year-old hypertensive male presented with features of acute coronary syndrome and planned for percutaneous transluminal coronary angioplasty of RCA followed by dual antiplatelet therapy. Since he had aspirin allergy, desensitization was done using rapid desensitization protocol for which he responded well. This case highlights the importance of aspirin-desensitization in patients with aspirin allergy instead of choosing non-aspirin based antiplatelet agents.

## INTRODUCTION

Aspirin (acetylsalicylic acid, ASA) is an antiplatelet agent that inhibits its activation and aggregation via irreversible inactivation of platelet-dependent enzymes cyclooxygenase (COX) isomers: COX-1 and COX-2. Blockage of COX-1 inhibits the production of thromboxane, a potent stimulator of platelet aggregation, thereby exerting its antiplatelet action.^1,2^ Though hypersensitivity reactions to other nonsteroidal anti-inflammatory drugs (NSAIDs) are common, hypersensitivity to aspirin is a rare entity occurring among 1.88% of participants.^3^ Herein, we present a rare case of a double vessel disease (DVD) with aspirin hypersensitivity who underwent aspirin desensitization as per the ADAPTED registry before the procedure.

## CASE REPORT

We present a case of a 74-year former smoker and hypertensive male, with prior coronary artery disease who visited our outpatient clinic with complaints of a crescendo pattern of exertional chest pain (Canadian Cardiovascular Society angina class III) for three months. He also had exertional dyspnea but did not have palpitation, pedal edema, cough, fever, and weight loss. He had a past history of coronary artery bypass graft, left internal mammary artery (LIMA) to left anterior descending (LAD) artery, and right internal mammary artery (RIMA) to D1 branch of LAD seven years back. He was regularly taking his prescription medicines i.e., clopidogrel, atorvastatin-ezetimibe, metoprolol, and losartan-amlodipine. He had aspirin hypersensitivity and was prescribed clopidogrel monotherapy after he developed severe urticarial rashes and angioedema following his prior exposure to aspirin.

During this visit, his treadmill test (TMT) was positive and the coronary angiography revealed double vessel disease (diffuse 95-99% stenosis in ostio-proximal LAD and focal 90-95% stenosis in proximal right coronary artery (RCA)) with a normal Thrombolysis in Myocardial Infarction (TIMI) III flow in both graft vessels. The interventional cardiology team advised percutaneous transluminal coronary angioplasty (PTCA) of RCA and was then planned for aspirin desensitization in anticipation of dual antiplatelet therapy (DAPT) following angioplasty. He was admitted for aspirin desensitization using the rapid aspirin desensitization protocol used in the multicenter ADAPTED (Aspirin Desensitization in Patients with Coronary Artery Disease) registry.^4^

He underwent aspirin desensitization with six sequentially increasing doses of oral aspirin starting from 1 mg over 5.5 hours. As per the protocol, he was not pre-treated with any of the steroids, antihistaminics, and anti-leukotrienes. His blood pressure, pulse rate, and oxygen saturation were recorded every 30 minutes until the end of the procedure along with monitoring of respirTSry, mucocutaneoffiB naso-ocular, and systemic symptoms. He did not develop any undue changes in his vital parameters nor developed any symptoms of hypersensitivity.

Following the uneventful aspirin desensitization, he underwent PTCA to RCA the next day successfully. He was discharged from the hospital after four days of hospitalization. He was prescribed with aspirin 300 mg once daily and clopidogrel 75 mg twice daily for one month. At one month follow-up, his antiplatelet medications were adjusted to aspirin 150 mg once daily and clopidogrel 75 mg once daily with a plan to continue DAPT for 1 year. He was asymptomatic and tolerating aspirin well at six-months of follow-up after desensitization and angioplasty.

## DISCUSSION

Primary and secondary prevention of coronary artery diseases (CAD) includes the usage of antiplatelet agents including aspirin alone or in combination, which decrease the prevalence of CAD events in up to 33% of total cases.^1^ However, some patients do not tolerate aspirin or develop hypersensitivity reactions to it. Aspirin intolerance is defined as either a proven hypersensitivity that manifests as aspirin-exacerbated respiratory tract disease, urticaria/angioedema, anaphylaxis, or severe indigestion following intake of low-dose aspirin.^1,5^

The definitive diagnosis of aspirin hypersensitivity is done by provocative aspirin challenge test via oral, bronchial, or nasal routes. However, in clinical practice, it is rarely done, and diagnosis is made on clinical grounds.^5^

Alternative therapy modalities, such as thienopyridines like clopidogrel, may be helpful in patients with aspirin hypersensitivity; nevertheless, aspirin desensitization appears to be more cost-effective than the usage of clopidogrel in these patients.^6^ In addition, following procedures like percutaneous transluminal coronary angioplasty (PTCA), patients require DAPT, which includes aspirin. In such situations, aspirin desensitization should be prioritized in aspirin-hypersensitive patients for maximal antiplatelet action instead of using non-aspirin-based combinations.^5^ There are multiple approaches for aspirin desensitization in ASA-hypersensitive patients undergoing PTCA.^4,7,8^ Of them, we adopted a rapid ASA desensitization protocol as per the ADAPTED registry. According to this protocol, six consecutive doses of aspirin (1, 5, 10, 20, 40, and 100 mg) are given orally for 5.5 hours.^4^

In a multicenter, observational study including 330 patients, adverse effects of aspirin included urticaria (53.6%), angioedema (20.9%), asthma (19.7%), and anaphylactic reaction (5.8%). The desensitization procedure was successful in 315 patients (95.4%) and in all the patients with a history of anaphylactic reaction.^4^ Similarly, another observational study including 24 patients depicted that aspirin desensitization was successful in 92% of them.^9^ A pooled desensitization success rate was found to be 98.3% in a meta-analysis among 480 patients with an acute coronary syndrome with a higher success rates for protocol using more than 6 dose escalations (as done in our case). Similarly, no hypersensitivity adverse events were reported on follow-up in these patients similar to our case.^10^ These studies showed that a rapid standardized protocol is safe and effective in patients with coronary artery disease, irrespective of the type of sensitivity. Therefore, in ASA-hypersensitive patients requiring DAPT, aspirin desensitization should be preferred instead of using non-aspirin-based DAPT for cost-effectiveness and maximal antiplatelet action.
